# Relative predation intensity of an intertidal gastropod on artificial coastal defense structures

**DOI:** 10.1002/ece3.11385

**Published:** 2024-05-12

**Authors:** Hannah H. J. Yeo, Jing Ying Yeo, Peter A. Todd

**Affiliations:** ^1^ Department of Biological Sciences National University of Singapore Singapore Singapore

**Keywords:** *Nerita undata*, predation risk, predators, seawalls, tethering, urban ecology

## Abstract

Despite seawalls becoming ubiquitous coastal features, and having some physical similarities to natural rocky shores, it remains unclear how these urban habitats influence predator–prey interactions. Predators can affect intertidal mobile prey densities through two pathways: (1) successful predation directly influences prey mortality rates, and (2) direct and indirect effects of predation can scare and induce motile prey to seek safer areas. In this study, we investigated whether intertidal predators affect the density of the marine gastropod, *Nerita undata*, at four seawall sites in Singapore. Using a tethering method that we developed, we monitored the mortality and other evidence of predation (shell state) of tethered *N. undata*. Field experiments revealed high (22.5%–82.5%) predation potential of *N. undata* across the four sites, with significantly higher predation risk at lower shore heights and for snails with mixed shell coloration. Observations and analysis of the shell state after 3 days showed that predation on seawalls was primarily by crushing predators such as fish. Other predators of *N. undata* include predatory snails, with various feeding methods that left behind different predator signatures. Our results add substantially to the limited knowledge on predator–prey interactions on seawalls, particularly for *Nerita undata*, and suggest that seawall systems are more dynamic than previously thought. This further highlights the role of these artificial structures as important habitats and feeding grounds in urban coastal ecosystems.

## Graphical abstract

In this study, we investigated whether predators affect the density of a common marine gastropod (*Nerita undata*) on tropical seawalls using a tethering method that we developed. Field experiments revealed high predation intensity, with a significantly higher predation risk at lower shore heights and for snails with mixed shell coloration. Our results add substantially to the limited knowledge on predator–prey interactions on artificial coastal structures, and identifies the key predators of *N. undata* (fish and predatory snails).

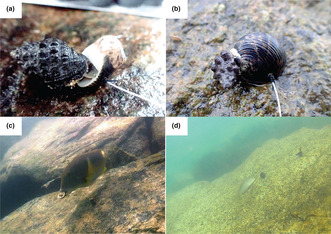

## 1 INTRODUCTION

Shorelines globally are undergoing extensive modification, especially the construction of artificial structures such as seawalls, breakwaters, and groynes (Bulleri & Chapman, 2015). The proliferation of these coastal defense strategies is a response to rising sea levels and increasing storm frequency due to climate change (Bloom, 2011; Gerland et al., 2014) and are especially prevalent near coastal cities that are expanding rapidly as more people migrate from rural to urban areas (Creel, 2003; Sengupta et al., 2018). Striking examples are Singapore, Taiwan, Hong Kong, Okinawa (Japan), and Penang (Malaysia) where hard defenses now comprise major portions of their coastlines (Chee et al., 2017; Lai et al., 2015; Lam et al., 2009; Masucci & Reimer, 2019; Yang et al., 2010). Although seawalls, especially the granite rip‐rap revetments common in Southeast Asia, and rocky shores are morphologically similar in that they are both sloping, hard‐surface, intertidal habitats, they also exhibit several fundamental differences that have important implications for their ability to support biodiversity. To effectively dissipate wave energy while using minimal resources such as materials and land space, large granite boulders are installed at high slope angles, but this leads to a lack of surface heterogeneity, water retaining features, and micro‐habitats that would normally be found on a natural shore (Firth et al., 2013; Loke et al., 2015). Furthermore, granite has high solar absorption and thermal conductivity, leading to high local temperatures (Coombes & Naylor, 2012; Sharma, 2017; Zhao et al., 2019). Due to these structural differences, artificial coastal defenses tend to support low‐diversity and different community assemblages than natural rocky shores (Firth et al., 2013; Moschella et al., 2005) and are often dominated by certain species (Chapman, 2006; Chapman & Bulleri, 2003; Ravinesh & Bijukumar, 2013). However, relatively little is known about mechanisms driving species diversity and abundance on these artificial structures.

Predation is one of the major evolutionary forces that shapes the structure and dynamics of ecological communities (Glasser, 1979) and is well documented in many intertidal and marine systems (e.g., Anderson & Connell, 1999; Karlson, 1978; Menge & Sutherland, 1976; Paine, 1974; Trussell et al., 2003). Since it was first introduced by Clarke (1962), the concept that predators can influence prey abundance and promote species diversity has been a key principle in community ecology. Many studies have since demonstrated that predation drives top‐down regulation, preventing prey populations from monopolizing resources and dominating the ecosystem, thus increasing abundance of less competitive species, promoting species diversity and species coexistence, especially in marine systems (see Connell, 1972; Freestone et al., 2011; Kremer & da Rocha, 2016; Lavender et al., 2017; Menge & Sutherland, 1976; Paine, 1966, 1974). Classic research from the 1960s and 1970s showed that higher species richness on rocky shores off the western coast of North America was due to strong predation effects exerted by the seastar *Pisaster ochraceus* on populations of the mussel *Mytilus californianus* (Paine, 1966, 1974). In contrast, the absence of predators or their reduced effectiveness can allow a handful of prey species to dominate in harsher (i.e., more disturbed) environments, resulting in reduced species diversity (Connell, 1972; Menge & Sutherland, 1976). Nevertheless, under certain circumstances, the presence of predators may reduce prey species abundance and richness, for example, when predation exceeds a threshold that results in reduced abundance/biomass and eventually removal of some prey species (Chang & Todd, 2023; Katano et al., 2015). Therefore, an understanding of predation or, more specifically, predation intensity, is usually essential to providing insights on the regulation and structuring of marine communities, particularly for motile intertidal species.

Predators can affect intertidal motile prey densities through two pathways: (1) successful predation directly influences prey mortality rates, and (2) direct and indirect effects of predation can cause fear and induce motile prey to seek safer areas (Preisser et al., 2005; Werner & Peacor, 2003). In intertidal rocky shore systems, predation is known to increase toward lower shore levels (Connell, 1972; Menge, 1978a; Rochette & Dill, 2000; Yamada & Boulding, 1996). Predators within groups such as seastars, gastropods, and crabs limit the distribution of sessile invertebrates (e.g., mussels and barnacles) and motile herbivorous gastropods directly through mortality from predation (Connell, 1972). For motile prey, the relationship between spatial variation in predation risk and prey distribution should not be automatically interpreted as a direct consequence of high mortality rates leading to low prey densities. This high predation risk can scare prey and cause them to change their behavior, for instance, actively moving to low‐risk habitats (Fawcett, 1984; Garrity & Levings, 1981; Rochette & Dill, 2000).

At present, it remains unclear how artificial coastal defenses—as habitats—influence predator–prey interactions and, by extension, community structure and biodiversity. In some cases, these human‐made structures could strengthen the trophic interactions between predators and prey, for instance, they may promote the colonization of algae communities—which then attract motile grazers and subsequently predators as a result of higher prey and food abundances (Ferrario et al., 2016; Jackson et al., 2008; Munsch et al., 2014; Pickering & Whitmarsh, 1997). This is supported by Munsch et al. (2014) and Ng et al. (2021), who identified greater fish foraging rates on seawalls compared to adjacent natural habitats. In other cases, artificial coastal structures may weaken trophic interactions by acting as barriers that limit access to foraging grounds and therefore restrict predator movement (Bishop et al., 2017).

Snails from the genus *Nerita* are one of the most common herbivorous gastropods on Singapore's intertidal coastal defenses (Lee & Tan, 2009), with an estimated population density over 50 times greater on seawalls than on adjacent rocky shores (Yeo, 2024). Individuals can grow up to 4 cm, and in large numbers this species represents a substantial biomass, making it one of the most dominant motile invertebrates in Singapore's seawall community. Previous studies also indicate *Nerita* spp. is well adapted to the harsh conditions found on tropical seawalls, particularly their ability to effectively regulate body temperature in the high intertidal zone (Chan et al., 2022; Vermeij, 1971). However, there are gaps in understanding relating to how predation influences their prevalence and distribution on seawalls or vice versa (i.e., how prey abundance influences predation).

There exists an extremely limited amount of scientific literature on the predation of *Nerita* spp. snails in the region (but see Bertness et al., 1981; Garrity & Levings, 1981; Garrity et al., 1986; Reynolds & Reynolds, 1977; Tong, 1986). The most relevant is a study of the natural rocky shores at St John's Island, Singapore (Chim & Ong, 2012), where field observations were performed to record direct evidence of predation events by an intertidal predatory snail, *Semiricinula fusca* (previously known as *Morula fusca*), which was later supported by stable isotope analysis (Lai et al., 2018). Such field observational studies of natural intertidal predation events are valuable, but challenging to achieve due to the time and resources needed to catch events that can span a few seconds to minutes and often occur underwater. An alternative approach is to tether the prey species and monitor mortality. This is usually achieved using epoxy to attach monofilament fishing line to the shell of motile organisms (e.g., Rochette & Dill, 2000; Yamada & Boulding, 1996). In the current study, we used a combination of field surveys and tethering experiments to investigate predator–prey interactions on the heavily modified coastlines of Singapore using *Nerita undata* as the focus prey species. We developed an effective and efficient tethering method that allowed us to restrain and monitor direct and indirect predation on *N. undata* by assessing predator traces left behind after 3 days and through recording predation attempts on GoPro video footage. The main aims of this field experiment were to (1) quantify predation intensity of *N. undata* on seawalls, and (2) examine the relationship between predation intensity and a suite of relevant variables (i.e., site, shore height, shell color, and shell size).

## 2 METHODS

### 2.1 Study sites, snail distribution, and size

**FIGURE 1:**
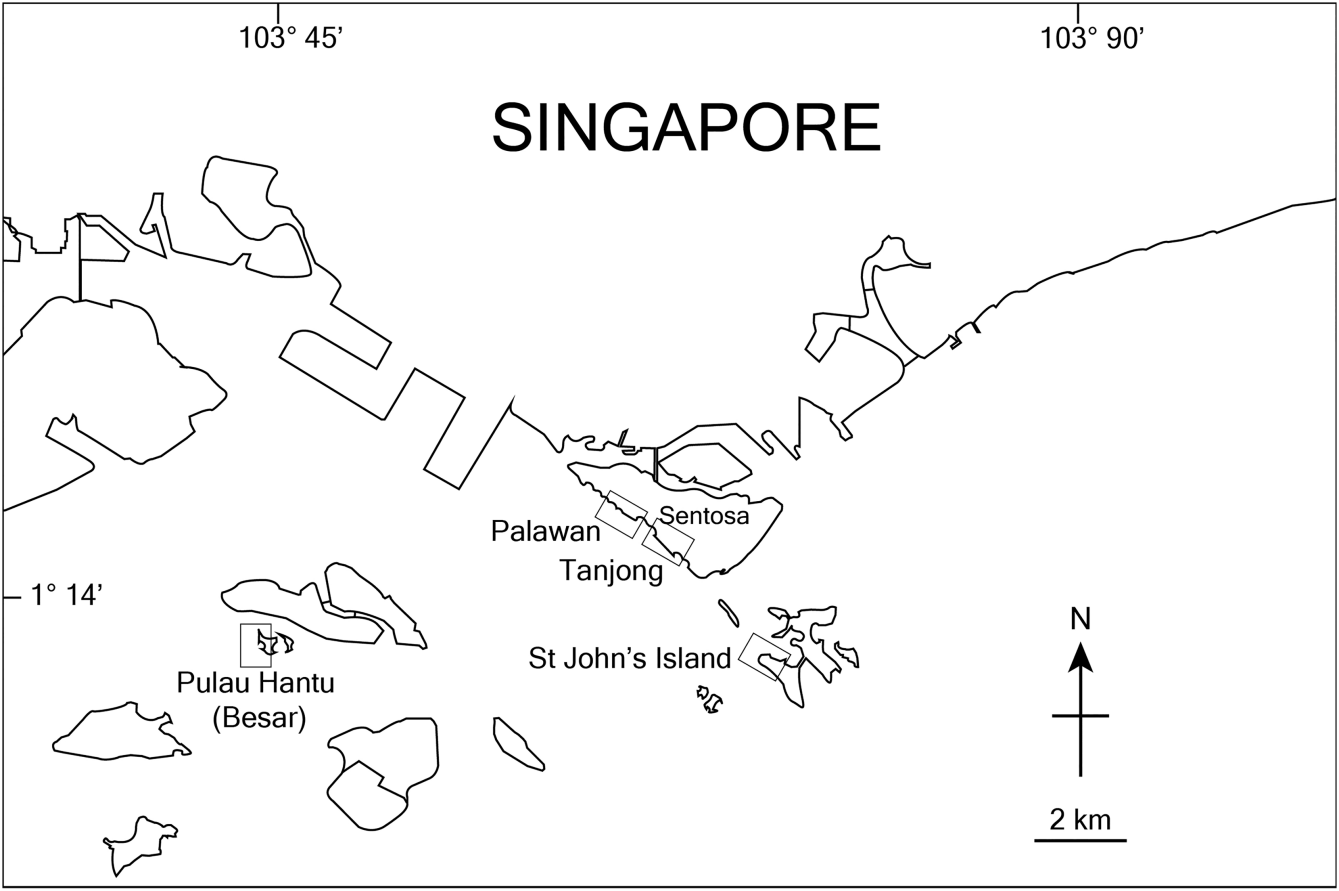
Location of the four study sites within the Southern Islands of Singapore.

The study was conducted at four sloping, rip‐rap, granite seawalls on three islands situated within the Southern Islands of Singapore (Figure 1); Palawan and Tanjong on Sentosa Island, Pulau Hantu (Besar), and Saint John's Island (hereafter termed as “St John's”). Each site was considered independent and sufficiently isolated or far apart from one another (i.e., at least 1.3 km apart and separated by sandy beaches or sea). Prior to the tethering experiments, we conducted site surveys to determine the vertical distribution, density, and shell sizes of *Nerita undata*. We focused on the sections of the seawalls that extended from ~0.6 m (a reasonably frequent low tide height) to 3.0 m (high spring tide) above chart datum. At each site, we positioned six vertical line transects, which were randomly selected along a 30 m length that stretched laterally across the wall. For each transect, we sampled 12 quadrats (50 cm × 50 cm) at increments of 50 cm up the sloping wall, starting from the base of the wall, with quadrats sequentially alternating between the left and right sides of the transect line. We estimated the tidal height of each quadrat by combining trigonometric approaches with hourly tidal height predictions from tidal stations and slope angle for each site. All snails found within the quadrats were photographed next to a scale bar and maximum shell length was measured using ImageJ.

### 2.2 Tethering

**FIGURE 2:**
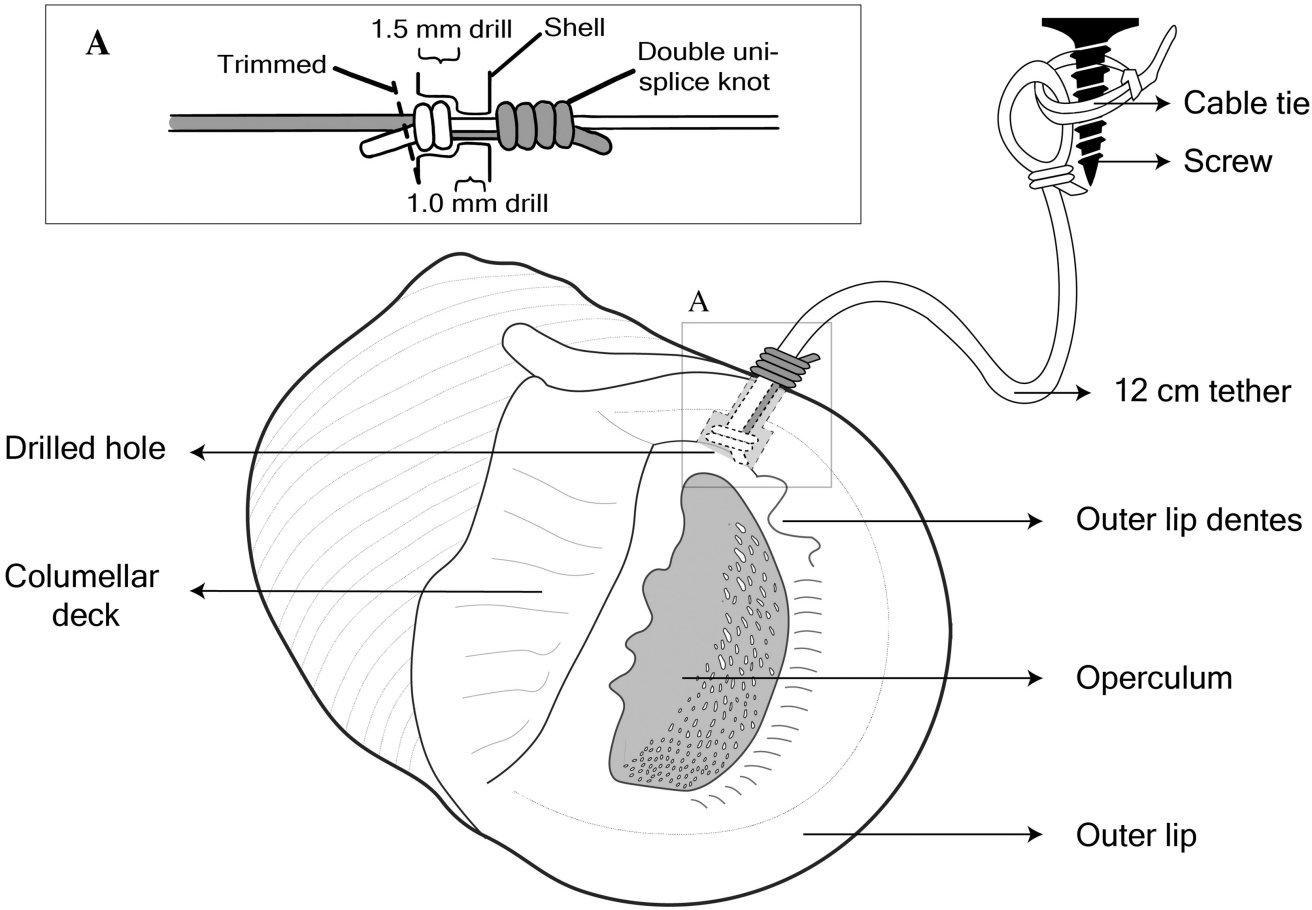
Diagram depicting the tethering method. Inset (A) showing the position where a hole was drilled in the outer lip of *N. undata*, which was attached to the 12 cm tether by a double uni‐splice knot.

To securely attach a line to a snail, we tested various tethering combinations of strings (natural and artificial threads, fishing line, etc.) and adhesives (epoxies, cyanoacrylates, etc.), but none were effective. Ultimately, we threaded a 0.5 mm transparent nylon monofilament through a drilled hole in the outer lip of the shell and knotted it (Figure 2). Prior to drilling, snails were dabbed dry until the snail retracted into the shell fully (i.e., with a closed operculum). A 1.5 mm drill bit was used to drill half‐way through the shell lip (between the columella deck and outer lip dente) and a 1.0 mm bit was used to drill through the rest of the shell. The use of two different sized drill bits created a recess for the knot so that it did not block the operculum from opening. Two monofilament strings with thickness of 0.5 mm (transparent, main line) and 0.38 mm (black, secondary line) were then threaded through the hole and a double uni‐splice knot (Figure 2A) was tied to secure the shell between two tight knots, rendering no need for the use of adhesives that may introduce tethering‐related artifacts or handling‐related biases in the experiment. At the end of the 0.5 mm monofilament, a loop (i.e., rapala knot) was tied. All the above tethering preparation work was conducted in the laboratory. In the field, the tethering point was a black 38 mm long screw that had been two‐thirds screwed into a plastic wall plug; that had been previously inserted into a 6.5 mm diameter hole drilled into the granite using a cordless hammer drill. A cable tie was then passed through the loop at the end of the monofilament and tightened around the protruding screw to the point that it was unable to slip over the screwhead, but was loose enough to spin freely around the screw (Figure S1); the “tail” of the cable tie was then trimmed off. Final distance from the screw to the snail was 12 cm.

A possible confounder in this study was the loss of snails due to non‐predator related causes, such as natural death, wave action, escape from the tether, or death from handling (Rochette & Dill, 2000). Therefore, we conducted control trials to experimentally assess induced mortality biases (see Arsenault & Himmelman, 1996; Barbeau & Scheibling, 1994; Rochette & Dill, 2000). For this, we tethered six snails on the seawall at Tanjong at both low‐ and high‐intertidal levels in stainless‐steel mesh (13 mm) predator‐exclusion cages (24 cm × 24 cm × 5 cm). In addition, we tethered another 12 snails in circular enclosures (24 cm diameter) with seawater in the laboratory. After 3 days, we noted whether the snails were (1) alive or dead, and (2) still attached to their tethers. Since all control snails (in situ and ex situ) were recovered alive and still attached to their tethers, we assumed that any dead and missing snails at the end of a 3‐day tethering experiment (see below) were due to predation.

### 2.3 Tethering experiment

**FIGURE 3:**
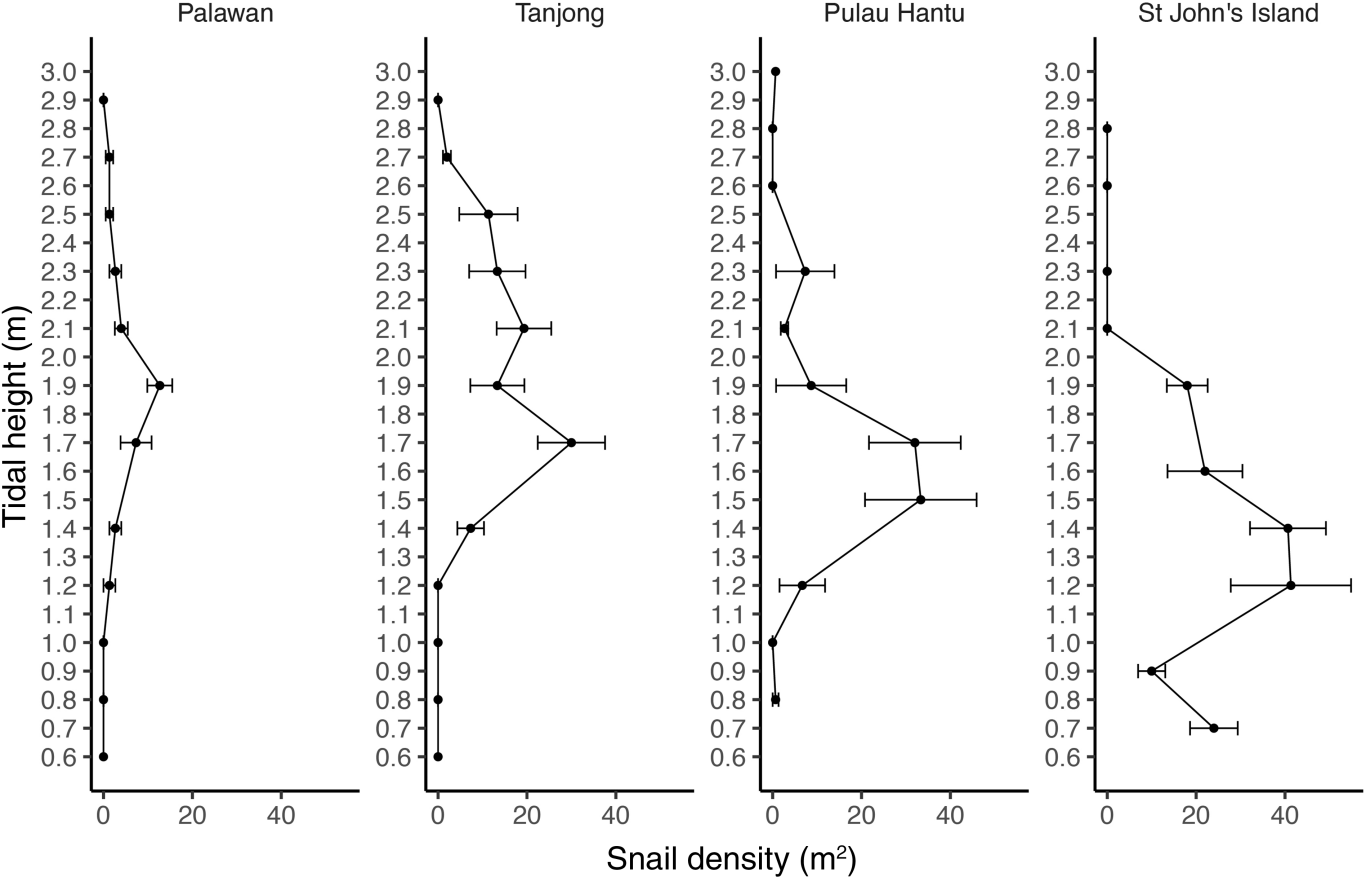
Mean snail density (±SE) of *N. undata* snails surveyed at different tidal heights in each study site.

Tethering experiments were conducted to determine whether *N. undata* predation intensity was different between low and high shore heights and among sites. Mortality rates of snails tethered at the low shore height (1.2 m above chart datum), near the lower end of their distribution (see Figure 3, Results), were compared to those tethered at the high shore height (2.1 m above chart datum), near the upper end of where this species was most abundant (see Figure 3, Results). For each experimental round (i.e., temporal replicates), snails of size length 21–24 mm were collected within 30 m of the study site. Snails were brought back to the laboratory, attached to a monofilament tether as described in Section 2.2 above, and left in seawater overnight. The following day, 40 individuals (i.e., 20 at high shore and 20 at low shore) were tethered at least 1 m apart from one another. During each experimental round, a control (one snail tethered within a 24 cm × 24 cm × 5 cm stainless‐steel 13 mm mesh predator‐exclusion cage) was placed concurrently at each shore height. All snails were photographed in situ with a scale bar and size (i.e., maximum shell length) was measured in ImageJ. Shell color was visually assigned by author HHJY into two categories: mixed and dark. Shells with white to light brown areas and dark/black blotches were classified as “mixed,” while those almost or fully black were classified as “dark” snails. After 3 days, we recorded whether snails were dead or alive, and photographed the condition of the shell. Snails that were recovered alive were cut off their tether and released. The experiment was replicated three times at 2‐week intervals for each site, resulting in a total of 504 tethered snails (including 24 controls). We did not have to adjust for broken tethers or non‐predatory mortality, as there were none found for all the pre‐experiment control trials and for the controls during the experiment runs.

We also tethered an additional 28 snails at both high and low shore and across the sites and recorded them with 28 GoPro cameras for ~4 h each. Video recordings were mostly started during daytime rising tides to enable the capture of predation events during high tides.

### 2.4 Statistical analysis

Statistical analyses and plot building was performed in R version 4.0.5. Mean percentage mortality of gastropods (i.e., the amount of dead snails as a percentage of the total number of tethered snails) was calculated for every combination of site, shore height, and experimental rounds. To compare predation intensity between the two shore heights, we used generalized linear mixed effects models (GLMMs) with the binary outcome of snails (“alive” or “dead”) as the response variable, and site, shore height (“low” vs. “high”), shell color (“mixed” or “dark”) and shell size as explanatory variables in a saturated model. Experimental rounds (i.e., temporal replicates) and plot identity were inputted as random factors in the model as tethering snails at the same sets of plots (40 plots at each site) on different experimental runs were not independent from each other. Prior to analysis, all continuous variables (i.e., shell size) were rescaled using the *rescale* function in the package *arms* (Gelman et al., 2021). Saturated models with interactions between all four main effects were created using a binomial distribution using the *glmer* function in *lme4* package (Bates et al., 2014). Akaike information criterion values were used to simplify and select the final and best model (Table S1), and the model fit was checked.

Shell condition of dead snails after 3 days were assessed and grouped into four mutually exclusive categories: (1) crushed, (2) empty shell, (3) pulled off tether, and (4) shell with hole, and plotted out in a Principal Components Analysis (PCA) biplot to visualize how shell condition varied across sites and at different shore heights. To investigate the variation in shell condition, we fitted GLMMs to the data set using the *MCMCglmm* package (Hadfield, 2010). Snail condition was modeled as a categorical variable with five levels (“alive,” “crushed,” “empty shell,” “pulled off,” and “shell with hole”). Because of non‐independence in plot locations and temporal replicates, we set a prior that had a variance of 1 and 0 for the covariance and fitted three models with main effects and interactions among shore height, site, shell color, and size. The three models were fitted to an ordinal family distribution and only differed with respect to the random variables that were included. A full model containing effects for both plot and experimental rounds was compared to models with only plot or rounds included as random variables. These models were run for 40,000 iterations, 8000 burn‐ins and the best‐fit model was selected using the deviance information criterion (DIC), with the lowest score model considered the best. Each model was run twice to minimize choice of wrong model due to Monte Carlo error in calculating DIC (Table S2) and trace sample output of the final model were checked.

## 3 RESULTS

All field surveys were completed successfully. We were able to find and recover the screws, cable ties, and monofilament lines used for all 480 tethered snails. All 24 tethered snails within the controls (predator‐exclusion cages) were recovered alive and in no case did the snails pull themselves free from the tether.

### 3.1 Snail distribution, density, and shell length

The field surveys revealed similar intertidal distribution and size structure of *Nerita undata* at the four study sites. *N. undata* were mostly absent below the 1.0 m tidal level, except at St John's (Figure 3). Across all sites, maximum snail density was observed within the 1.2–1.9 m tidal range and there was a decline in abundance up the shore until snails were absent beyond 2.7 m above chart datum (Figure 3). *N. undata* within the size length 15–21 mm were the most abundant at all four sites (Figure S2). Smaller snails with shell between 15 and 21 mm were observed more frequently at Palawan and Pulau Hantu, while larger snails with shell length between 21 and 27 mm were more abundant at Tanjong and St John's (Figure S2). Across all four sites, the shell size of *N. undata* generally decreased with tidal and shore height (Figure S3).

### 3.2 Tethering experiment

**FIGURE 4:**
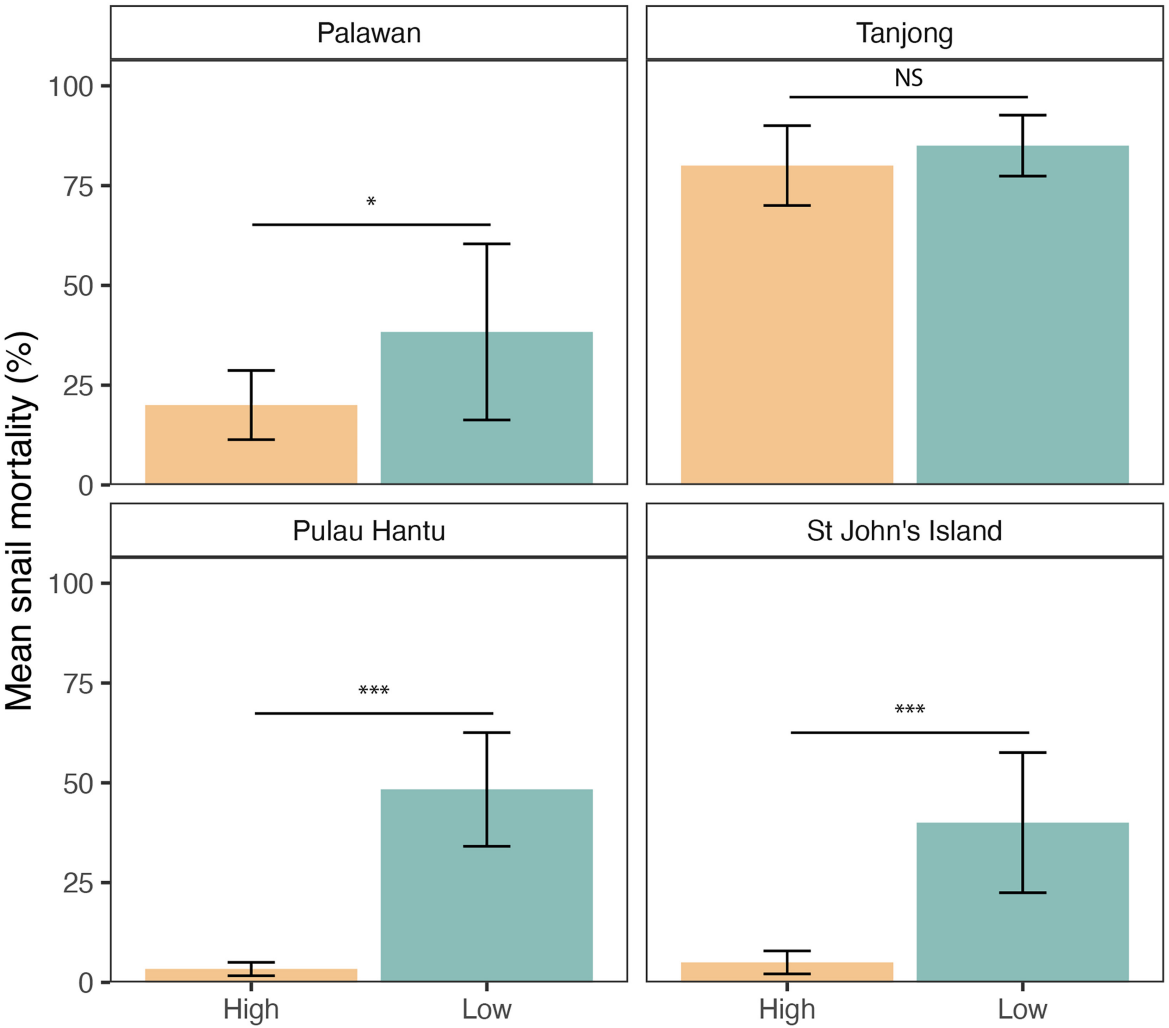
Mean (±SE) percentage mortality of tethered *N. undata* that was eaten by predators after 3 days at low and high shore heights at each study site (*p < .05, ***p < .001, NS = not significant).

Overall, tethered snail mortality over the whole experiment was 40%. Among all the sites, average snail mortality was highest at Tanjong (82.5%), followed by Palawan (29.2%), Pulau Hantu (25.8%), and lowest at St John's (22.5%) (Figure 4). Mean mortality rates during the three experimental rounds varied between 5% and 80% for Palawan, 60%–100% for Tanjong, 0–65% for Pulau Hantu, and 0–75% for St John's (Figure S4). Most of this variability was related to a significant site effect (*p* < .05). Snails tethered in Tanjong experienced almost three times greater mortality compared to snails tethered at the other sites (Figure 4; Table 1). The variability in snail mortality was also explained by intertidal shore height (*p* = .030) and shell color (*p* = .033). *N. undata* was preyed on more frequently at the low shore height than those tethered at the high shore height; this difference was statistically significant at Pulau Hantu (*p* < .001), St John's Island (*p* < .001), and Palawan (*p* = .030), but not at Tanjong (Figure 4; Tables 1 and 2). Snails with “mixed” shell coloration were more frequently preyed on than “dark” snails (*p* < .050). Shell size had no effect on mortality (*p* = .582, Table 1).

**TABLE 1:** Simplified generalized linear mixed models with binomial distribution summary table for snail mortality (*p < .05, **p < .01, ***p < .001).

GLMM	Estimate	SE	*z* value	Pr(>|*z*|)
Intercept (Palawan, High, Dark)	−1.8254	0.4945	−3.691	0.000***
Site (Tanjong)	2.9945	0.4777	6.268	0.000***
Site (Pulau Hantu)	−2.0842	0.8061	−2.586	0.010*
Site (St John's)	−1.6240	0.7087	−2.292	0.022*
Shore Height (Low)	0.9308	0.4293	2.168	0.030*
Color (Mixed)	0.5292	0.2489	2.126	0.034*
Size	0.2828	0.5138	0.550	0.582
Site (Tanjong): Shore Height (Low)	−0.6727	0.6560	−1.025	0.305
Site (Pulau Hantu): Shore Height (Low)	2.5105	0.8822	2.846	0.004**
Site (St John's): Shore Height (Low)	1.7411	0.7842	2.220	0.026*

**TABLE 2:** TukeyHSD pairwise comparison between shore height for each given shore height using *emmeans* (*p < .05, **p < .01, ***p < .001).

Contrasts (emmeans)	Estimate	SE	df	*z* ratio	*p* value
Palawan (high–low)	−0.931	0.429	Inf	−2.168	.030*
Tanjong (high–low)	−0.258	0.496	Inf	−0.521	.603
Pulau Hantu (high–low)	−3.441	0.772	Inf	−4.457	<.001***
St John's (high–low)	−2.672	0.658	Inf	−4.063	<.001***

**FIGURE 5:**
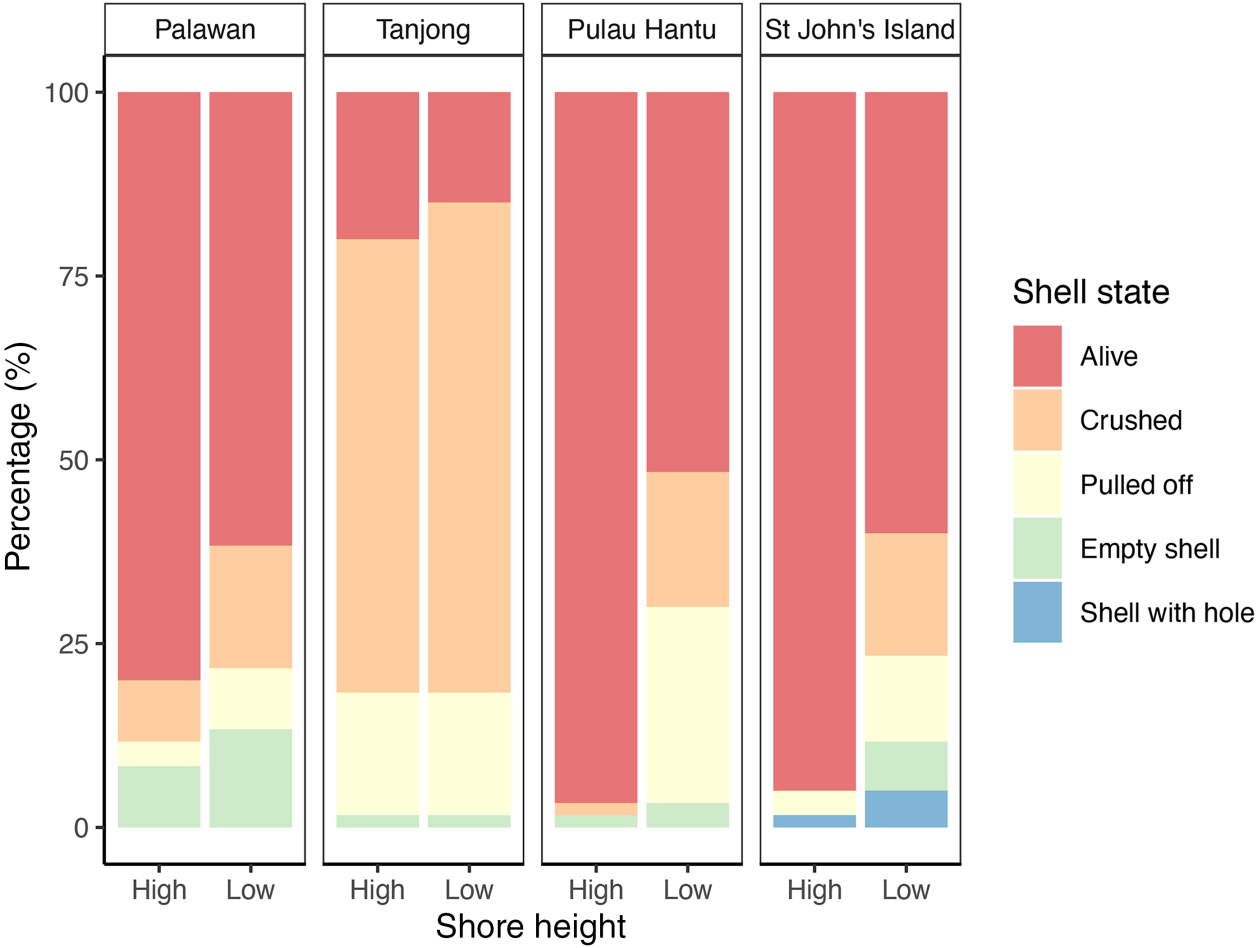
The relative percentage contribution of shell conditions for high and low shore heights at each site.

**FIGURE 6:**
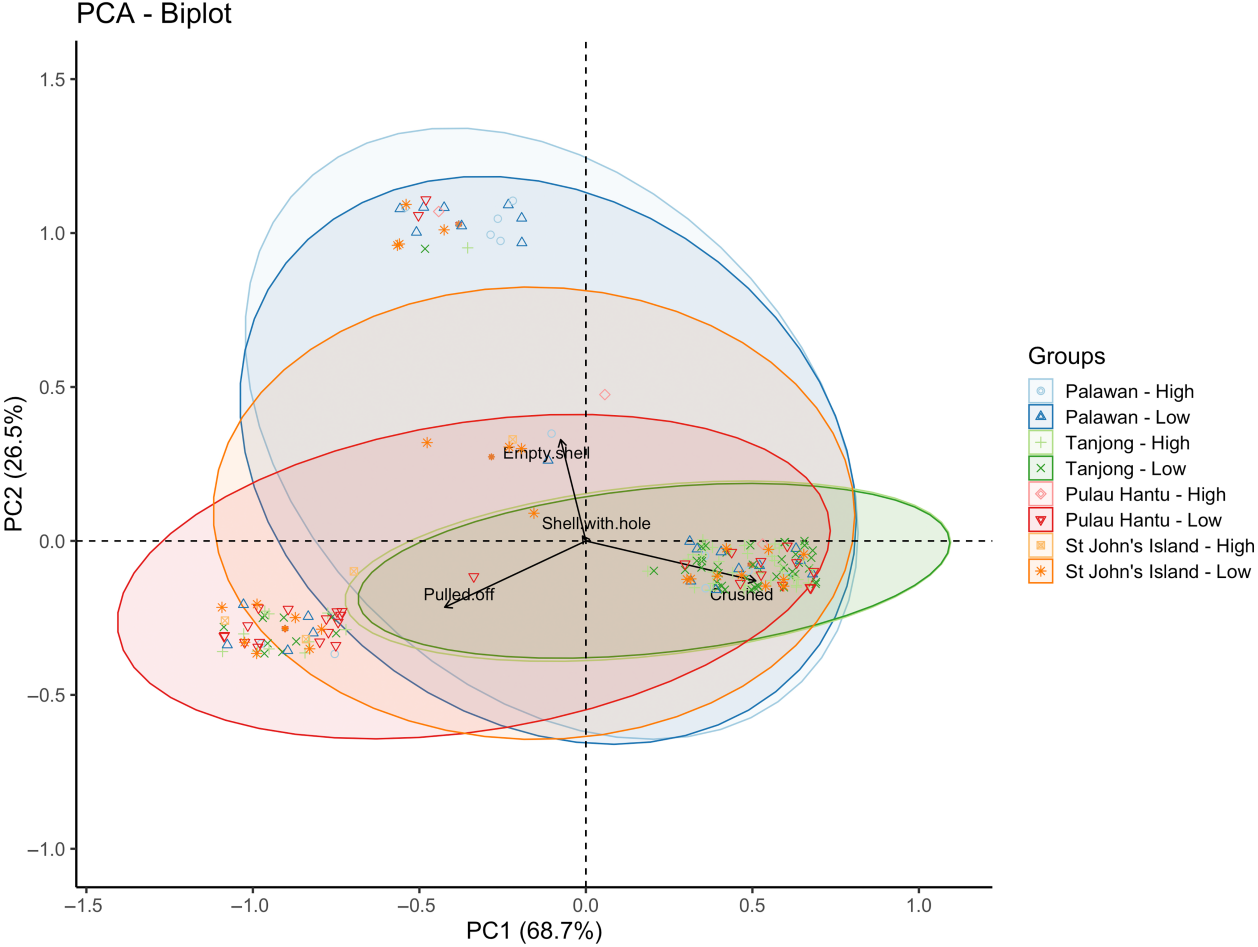
Principal Component analysis (PCA) plot illustrating shell states and clusters among the four sites and two shore heights. A confidence ellipsis at 70% has been drawn for each group. The first principal component explained 68.7% of variation and was most strongly associated with crushed shells, while the second principal component explained 26.5% of the variation and was most strongly associated with empty shells (Tables S3 and S4).

By the end of the experiment, the majority of snails were recovered alive (60.0%). The remaining 40.0% were presumed dead, and were recorded as crushed (23.8%), empty but with intact shells (4.6%), shells pulled off their tethers entirely (10.8%), and as shells with holes drilled by predators (0.8%) (Table 3; Figure 5). We did not observe any evidence of peeling (i.e., cracks or breaks in shell aperture, see Bertness & Cunningham, 1981). The only significant finding for shell condition after 3 days was an interaction between site, color, and shell size at St John's (Table S5). The majority of dead snails at Palawan, Tanjong, and St John's had shells that were crushed, whereas at Pulau Hantu the snails were mostly pulled off their tether (Table 3). St John's was the only site where holes in shells were observed (Figure 5). The principal component analysis also confirmed that the variability of snail shell state at the end of the experiment was greater among sites than between shore heights within each site (Figure 6). For all sites, no particular shell state was more prevalent at the high shore compared to the low shore and vice versa.

**TABLE 3:** Condition of all 480 tethered snails after 3 days.

Shell state	Palawan		Tanjong		Pulau Hantu		St John's	
	High	Low	High	Low	High	Low	High	Low
1. Alive	48	37	12	9	58	31	57	36
2. Crushed	5	10	37	40	1	11	0	10
3. Pulled off	2	5	10	10	0	16	2	7
4. Empty shell	5	8	1	1	1	2	0	4
5. Shell with hole	0	0	0	0	0	0	1	3
Preyed upon	12	23	48	51	2	29	3	24

*Note*: There were a total of 120 snails tethered per site and 60 snails per shore height.

## 4 DISCUSSION

Predator–prey interactions on artificial coastal structures are relatively understudied as compared to natural intertidal rocky shore systems (Koch et al., 2007; Menge, 1978a, 1983; Rochette & Dill, 2000; Yamada & Boulding, 1996). Given the central role of herbivorous gastropods as both the primary consumers and prey in coastal food webs, understanding predation rates on these gastropods is important. We identified high *Nerita undata* mortality across all four sites during our field experiments, with predation intensity significantly greater at lower shore heights and for gastropods with mixed shell coloration. Crushing was the primary mode of predation, with almost a quarter of all the snails found with their shells crushed at the end of the experiment.

Field experiments over a 3‐day period revealed that gastropod prey on seawalls sites in Singapore faced high predation potential. Overall mortality rate was 40%, and up to 82.5% at one site, which is more than that reported in several temperate rocky shore studies (e.g., 2%–40% *Littorina* spp. in Rochette & Dill, 2000; 10%–20% *Littorina saxatilis* in Pardo & Johnson, 2005; 38% for single *Littorina plena* in Koch et al., 2007), but not as high as Yamada and Boulding (1996) at 77% or Perez et al. (2009) at 87%. The high predation rates were somewhat surprising, given that previous studies found lower rates in artificial habitats as compared to natural habitats across latitudes (Rodemann & Brandl, 2017; but see Paxton et al., 2020). A possible explanation for this is the low wave energy that characterizes Singapore's shores, as consumer effect is generally inversely correlated to environmental stress (Menge & Sutherland, 1987). Stressful conditions such as wave exposure can restrict and limit the feeding ability and activity of motile predators (Menge, 1978a, 1978b). Further, higher abundance of crab predators in sheltered locations has previously been reported (see Silva et al., 2010). Heery et al. (2020) also showed *N. undata* was less abundant at the more sheltered seawall locations in Singapore.

Scientists have suggested two simplified models for determining trophic organization: the bottom‐up and the top‐down model. With the former, availability of nutrients, which heavily influence primary producers, exerts bottom‐up regulation of the abundance of herbivores and higher‐order consumers (Sinclair & Krebs, 2001). Whereas in the latter, predation by higher trophic levels exerts a top‐down regulation that limits the number of herbivorous, plants, and nutrient levels (Sinclair & Krebs, 2001). Given that *N. undata* is abundant on seawalls despite high predation intensity (which suggest high abundance of predators), seawall communities could be more heavily regulated by bottom‐up controls. However, to confirm this would require additional studies (e.g., herbivore–algae interactions).

Predation risk and intensity increases down the shore. The low shore areas were on average submerged 77% of the time compared to 45% of the time for the high shore areas and predation risk of snails is likely tied to immersion duration, as has been shown in multiple studies across a wide range of rocky intertidal habitats. Many motile predators of gastropods (e.g., crustaceans and fish) undertake foraging excursions into the submerged intertidal zone. Predatory snails (e.g., whelks) are also usually restricted to the lower intertidal shores, or limited to foraging when covered by waves due to the risk of desiccation (Menge, 1978a). This greater predation potential probably explains why our field surveys revealed an absence of *N. undata* below the 1.0 m tidal level. In Singapore, high granite rock surface temperatures of up to 57°C (Chan et al., 2022) results in heat stress on the upper part of seawalls. There was a trend of snails higher on the seawall being smaller (Figure S3), even though they hold proportionately less water than larger snails for evaporative cooling and hence are more susceptible to heat stress (Garrity, 1984; Gray et al., 2005; Lowell, 1984). While behavioral responses to predation intensity were not evaluated, it is plausible that *N. undata*, especially the smaller individuals which are more susceptible to attack, migrate to higher areas on seawalls to avoid marine predators (Kurhe et al., 2014; McCormack, 1982; McQuaid, 1982). Hence, despite the greater food abundance and less harsh physical conditions at low‐intertidal levels, many species of gastropods may avoid such areas due to the presence of subtidal predators. Tanjong, however, differed from the other three sites by exhibiting high predation rates that were similar for both low and high shore level. This site was unusual in that shade from trees, relatively denser mats of turf and macroalgae, combined with deeper crevices in between boulders and more frequent wave splash from passing vessels may have provided damp refuges at the higher shore height for snail predators.

The primary cause of *N. undata* mortality was from crushing. In some instances, broken shell fragments remained on the tether, while other snails were completely crushed and/or pulled off the tether—which would be expected from predators such as crabs and fish. These motile predators and their consumption effect are relatively well documented on natural rocky shores (see Bertness & Cunningham, 1981; Edgell & Rochette, 2008; Menge, 1983; Robles et al., 1989; Silva et al., 2010). For instance, Menge (1983) found that shell crushing crabs had the highest per capita consumption rate compared to starfish or drilling gastropods. Although there are no records of crabs crushing *N. undata* in Singapore, previous studies have found that the fecal matter of an intertidal crab species, *Ozius guttatus*, that possesses large specialized chela that enables it to crush hard‐shelled gastropods (Fong, 2003, unpublished; Dietl & Vega, 2008), contained traces of *Nerita* sp. (Fong, 2003, unpublished). However, this decapod species was not commonly sighted around the seawall sites. There is also generally a lack of crab predators with a chela gape wide enough to seize or crush mollusks beyond a certain size (e.g., 20–26 mm see Ellis, 2000 and Han et al., 2008). Nerites have a very distinct shell morphology (e.g., short spire and globular in shape) that inhibits shell peeling by crabs (Bertness & Cunningham, 1981). Furthermore, shells of Indo‐West Pacific nerites have thicker walls, reduced spirals and more occluded apertures than East Pacific or West Atlantic *Nerita* species (Reynolds & Reynolds, 1977; Vermeij, 1976). These morphological adaptations, coupled with the globular and particularly smooth shell of *N. undata*, may increase their resistance to crab predation since it is harder for crabs to grip the snail and apply crushing force to a central area (Bertness & Cunningham, 1981; Hughes & Elner, 1979; Kitching et al., 1966; Kitching & Lockwood, 1974; Vermeij, 1976). Commonly sighted crabs on the sloping seawalls in this study included *Metopograpsus* spp., Sesarmidae spp., *Pilumnus vespertilio*, *Actaeodes* spp., *Lithoselatium kusu*, *Grapsus albolineatus*, and portunids (Personal observation.). These species, however, mostly feed on algae, detritus and/or small mollusks due to their relatively small chela, and are unlikely capable of crushing the tethered snails. It may be possible that large decapod predators migrate in and out of the seawall habitat between tides and day and night, making their numbers and potential predation effects difficult to assess.

**FIGURE 7:**
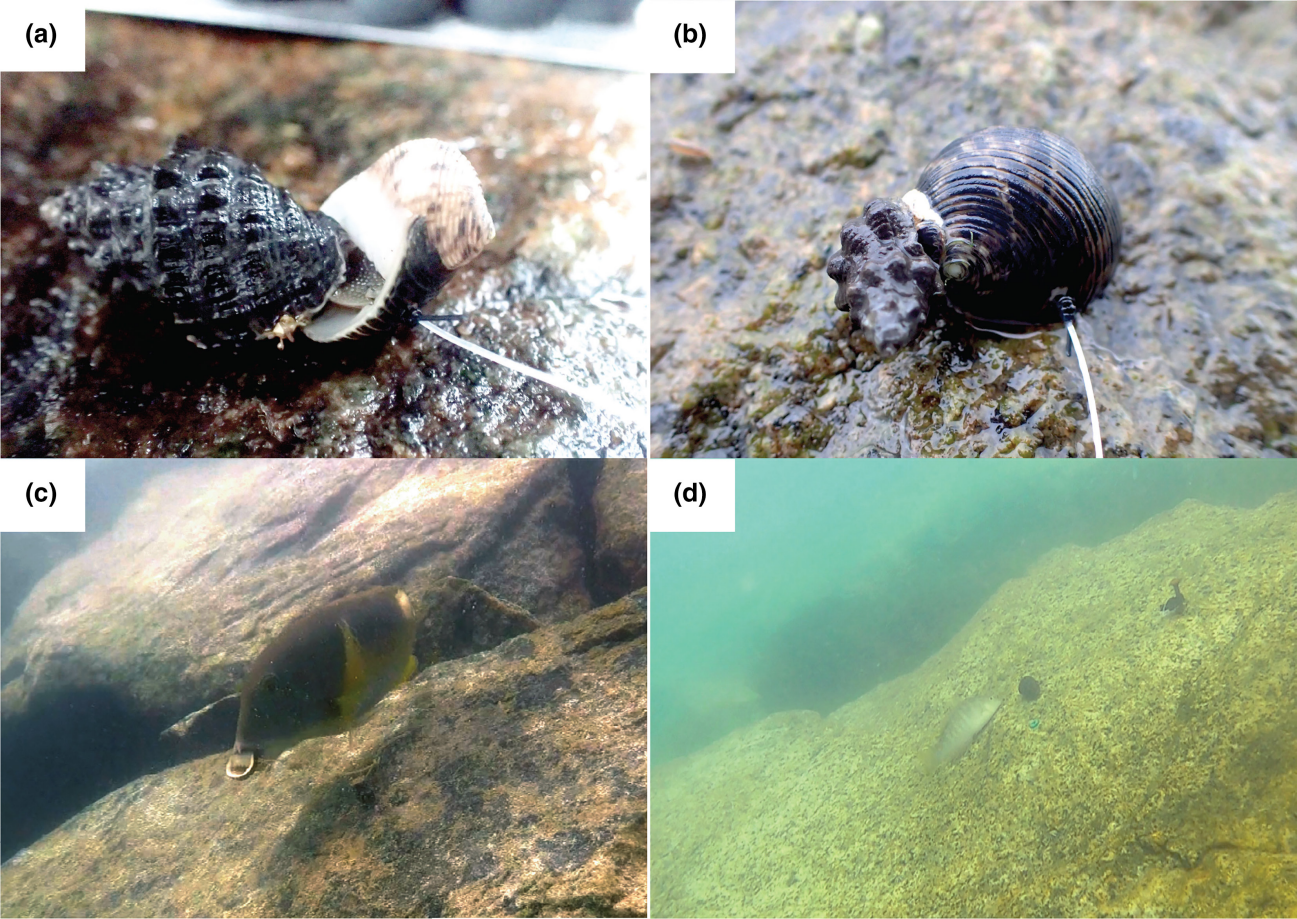
Evidence of predators feeding on tethered *Nerita undata*. (a) *Reishia clavigera* feeding through the operculum of prey, (b) *Murichorda rumphiusi* drilling a hole in the shell of prey, (c) *Choerodon anchorago* biting and pulling away tethered *N. undata* was seen on multiple occasions and (d) *Halichoeres nigrescens* attempting to bite a tethered snail through the underside of the shell.

Fishes use their mouths to crush gastropods, and the lack of knobs, spines and high spires decreases the effective diameter of *N. undata* shells, making them accessible to a wider range of fish sizes (Palmer, 1979). Fishes equipped with strong jaws and specialized dentition for crushing shells are more common in the tropics than in temperate systems (Palmer, 1979), and are potentially the predators responsible for the majority of crushed tethered snails in the current study. Possible fish predators on seawalls include the anchor tusk fish (*Choerodon anchorago*) and wrasse (*Halichoeres* spp.). However, despite observing tthese species biting and pulling tethered snails during high tide on multiple occasions in the video footage, (see Figure 7), the duration of the video in this study was insufficent to capture any instances of successful crushing. Interestingly, the lighter “mixed” shell color morphs were associated with higher predation rates, and this may explain why the darker nerite morphs tend to be more common in the field. Previous experiments using “Robonerites” found that shell coloration in nerites is probably not driven by temperature (Chan et al., 2019), suggesting other factors such as concealment from visual predators. Some fish can select gastropod prey based on coloration (e.g., the blenny *Blennius pholis*, Reimchen, 1979) and the lighter “mixed” shell color morphology of *N. undata* individuals could be more conspicuous against the gray granite seawalls.

The presence of both empty shells and shells with drilled holes suggest predation by carnivorous gastropods. Shell‐drilling gastropods from the Muricidae family (e.g., *Tenguella musiva*, *Drupella margariticola*, *Reishia* spp.) are common on seawalls in Singapore (Lee & Tan, 2009; Tan et al., 2018). We observed a predatory snail, *Reishia clavigera* feeding on the flesh of a tethered *N. undata* through the operculum (Figure 7), leaving behind an empty shell with the operculum still attached. We also recorded several instances of *Murichorda rumphiusi* (also commonly known as *Semiricinula fusca*) preying on tethered *N. undata* (Figure 7). Chim and Ong (2012) found *Semiricinula fusca* (previously *Morula fusca*) feeding on *N. undata* on the rocky shores at St John's Island and Lai et al. (2018) found isotopic traces of *N. undata* in tissue samples from *Drupella margariticola*, *Semiricinula fusca* and *Tenguella musiva* (previously *Morula musiva*). Other potential predators that are commonly seen on the seawalls include shore birds (e.g., herons, mynahs), rats, and monitor lizards (Personal observation), although there are no published records of them feeding on nerites. Susceptibility of gastropods to these aerial and terrestrial predators is likely to be greater for snails on the higher shore areas since birds and terrestrial predators can forage there for most of the day, but can only feed at the low shore when the tide is down. However, the majority of snail mortality in the experiments were at the lower shore height, suggesting the impact of these predators was limited. We used human observation extensively for daytime and night‐time above water surveys, and camera footage for daytime below water surveys, but rarely observed predation events. Based on this, we expect that the majority of predation events are occurring below water nocturnally, but capturing underwater footage of natural behavior at night requires specialized equipment that we do not have access to.

Beyond this study, future work could compare the predation intensity between natural and artificial hard intertidal habitats. Given the evidence of high predation intensity of *Nerita* on tropical seawalls, determining trait‐mediated interactions among predators, prey, and prey resources is key. More broadly, in addition to having a direct impact on the density of prey populations via consumption, predators also have non‐lethal indirect effects. For example, fear of predation can induce changes in behavioral (e.g., foraging strategies) and morphological traits (Abbey‐Lee et al., 2016; Richardson & Brown, 1992; Trussell et al., 2003). The Landscape of Fear (LOF) concept, which is defined as the spatial distribution of perceived predation risk by a prey population, is increasingly being regarded as the missing link to fully understanding trophic cascades (Bleicher, 2017; Laundré et al., 2001; Madin et al., 2011; Suraci et al., 2016). LOF is a measure of how prey species balance the trade‐offs between food and safety when foraging in a dangerous habitat. As such, LOFs has wide research applications, such as trophic dynamics, predation risk, energetic states, population demographics and size, density dependence (intraspecific competition), and community structure (interspecific competition). All of these are relevant to our study system and represent exciting research areas for the future.

Tethering experiments provide important insights into predation patterns and processes under field conditions (Aronson & Heck, 1995); however, they require careful interpretation. Importantly, mortality rates of tethered snails are inflated because the snails are confined to the rock surface and are thus unable to seek refuge from predators. Hence, mortality rates of tethered organisms should not be interpreted as absolute predation values of untethered prey (Aronson & Heck, 2001; Baker & Waltham, 2020). Nevertheless, comparative assessments are possible, and this tethering experiment enabled us to examine how site, shore height, shell color, and shell size can influence predation intensity of gastropods on artificial structures. We obtained new insights into the common and potential predators of intertidal gastropods (i.e., fish and carnivorous gastropods), leading to a better understanding of the physical and biological interactions occurring within intertidal communities living in these highly modified habitats. Our results suggest that artificial urban intertidal habitats may be more dynamic than previously thought and their role as habitats and feeding grounds in urban coastal ecosystems are potentially underestimated.

## AUTHOR CONTRIBUTIONS

**Hannah H. J. Yeo:** Conceptualization (lead); data curation (lead); formal analysis (lead); funding acquisition (supporting); investigation (lead); methodology (lead); project administration (lead); supervision (equal); visualization (lead); writing – original draft (lead); writing – review and editing (lead). **Jing Ying Yeo:** Conceptualization (supporting); data curation (supporting); formal analysis (supporting); investigation (equal); methodology (equal); project administration (supporting); visualization (supporting); writing – review and editing (equal). **Peter A. Todd:** Conceptualization (supporting); funding acquisition (lead); methodology (supporting); supervision (equal); writing – review and editing (equal).

## CONFLICT OF INTEREST STATEMENT

The authors declare no conflict of interest.

## Supporting information


Data S1

## Data Availability

All data that support the findings of this study have been made publicly available and can be accessed via FigShare. DOI: 10.6084/m9.figshare.23639838.
