# Supplementary material for: Relative predation intensity of an intertidal gastropod on artificial coastal defense structures
Source: Ecol Evol. 2024 May 12;14(5):e11385. doi: 10.1002/ece3.11385 (PMC11089086; doi:10.1002/ece3.11385)
Supplement: Supplementary file 1 — Data S1 [file ECE3-14-e11385-s001.docx]

*Supporting information*

**Relative predation intensity of an intertidal gastropod on artificial coastal defence structures**

Hannah H. J. Yeo^1,*^, Jing Ying Yeo^1^, Peter A. Todd^1^

^1^ Department of Biological Sciences, National University of Singapore, Singapore 117558

* Corresponding author: h.yeo@u.nus.edu;

Co-corresponding author: [dbspat@nus.edu.sg](mailto:dbspat@nus.edu.sg)


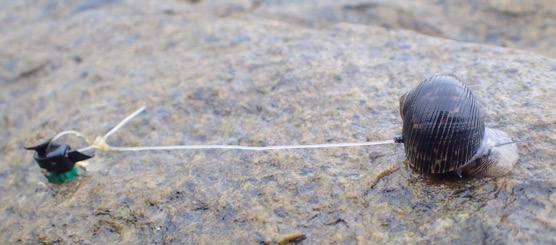


**Figure S1.** Individual *N. undata* tethered on seawall to a black screw using black cable ties.

**
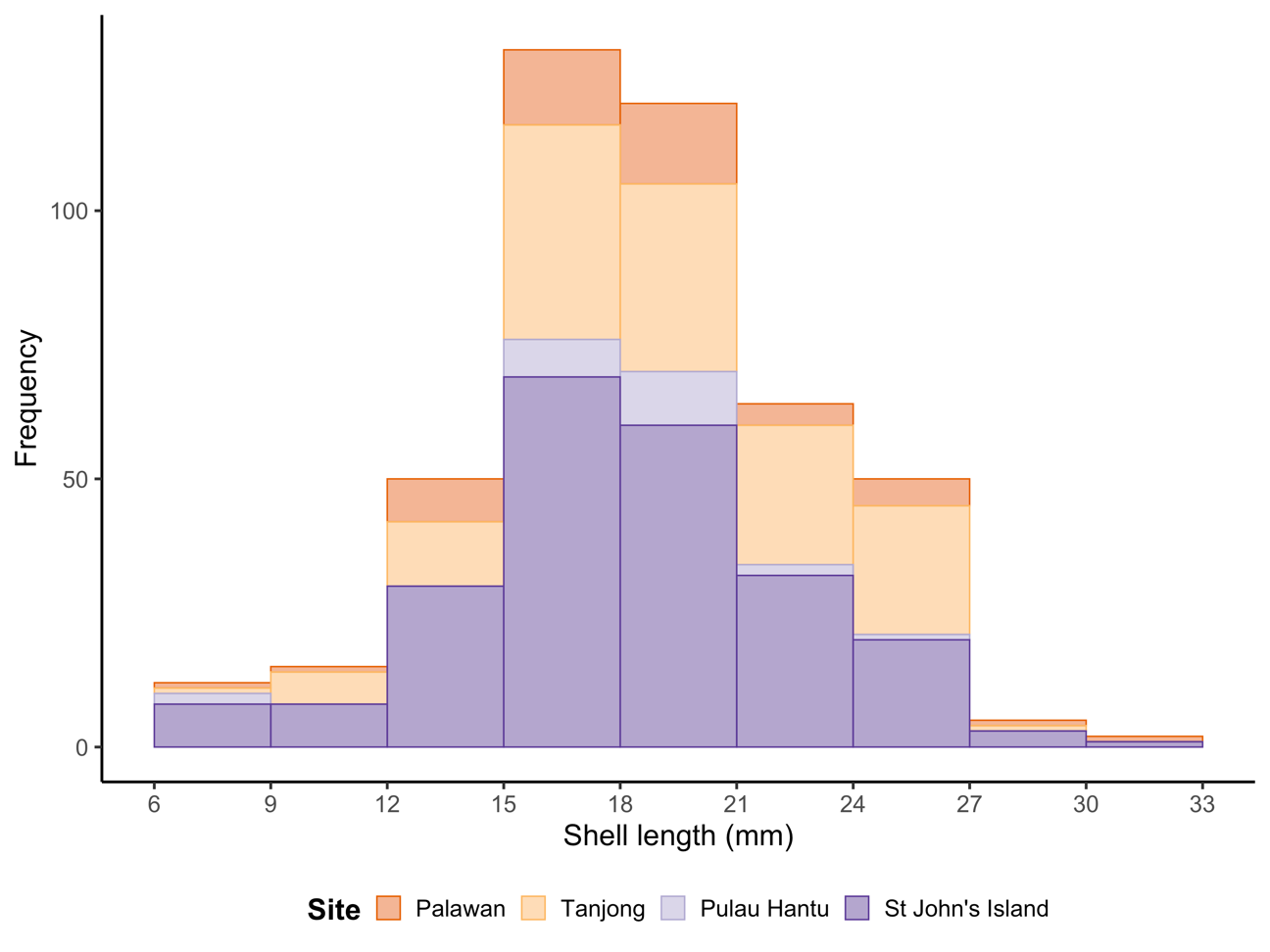
**

**Figure S2.** Size-frequency distribution for *N. undata* found at all four study sites.


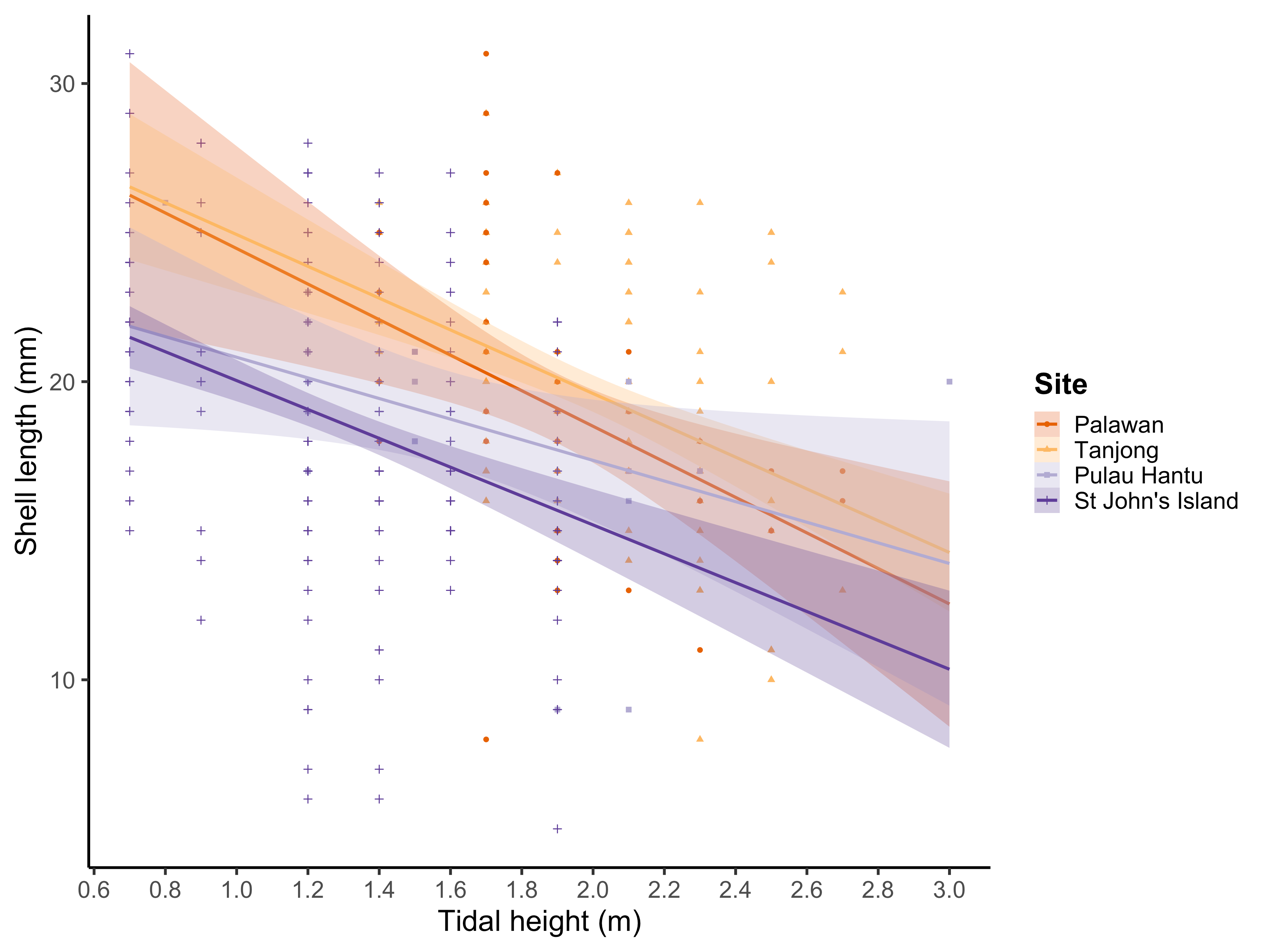


**Figure S3.** Shell size gradient with tidal height for *N. undata* found at all four study sites.

**
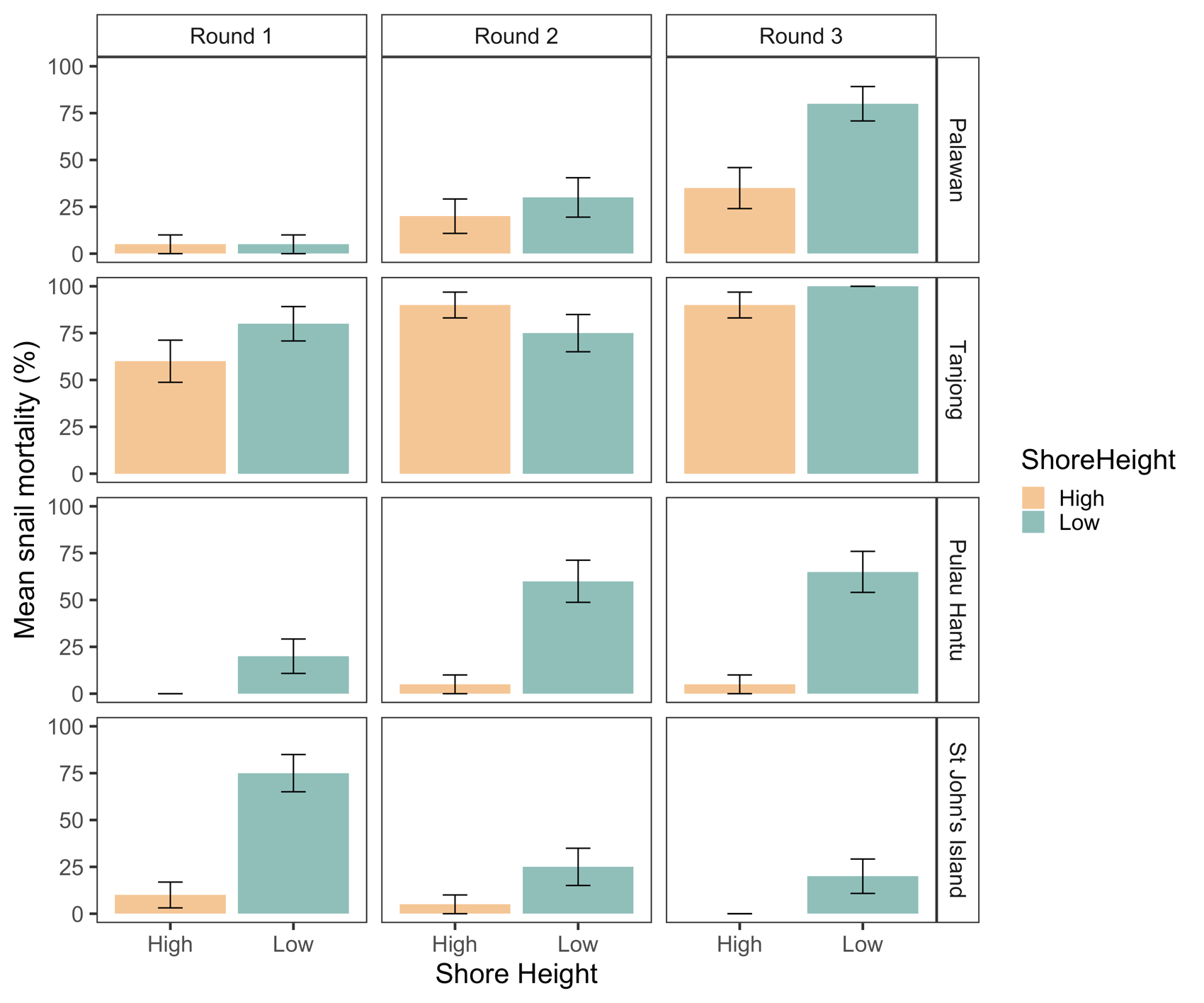
**

**Figure S4.** Mean (±S.E.) percentage mortality of tethered *N. undata* after three days for each experimental round and site.

**Table S1.** Generalized linear mixed effect models from most saturated to simplified model. *df* = degrees of freedom; AIC = Akaike information criterion values were used to simplify and select the final and best model.

| **No.** | **Model Formula** | ***df*** | **AIC** |
| --- | --- | --- | --- |
| 1 | Mortality ~ Site * Shore Height * Colour + Size + (1 \|Plot) + (1 \|Round) | 19 | 474.65 |
| 2 | Mortality ~ Site * Shore Height * Colour + Size + (1 \|Round) | 18 | 474.65 |
| 3 | Mortality ~ Site + Shore Height + Colour + Size + (1 \|Round) + Site : Shore Height + Site : Colour + Shore Height : Colour | 15 | 469.65 |
| 4 | Mortality ~ Site + Shore Height + Colour + Size + (1 \|Round) + Site : Shore Height + Site : Colour | 14 | 467.74 |
| 5 | Mortality ~ Site + Shore Height + Colour + Size + (1 \|Round) + Site : Shore Height | 11 | 465.03 |

**Table S2.** Deviance Information Criteria for several random effect models. Each model was run twice in order to assess the level of Monte Carlo error in calculating DIC.

| **Random effects in model** | **DIC 1** | **DIC 2** |
| --- | --- | --- |
| Full (Plot:Round) | 21.98145 | 27.90115 |
| Round | 334.91026 | 332.63678 |
| Plot | 303.37847 | 310.45353 |

**Table S3**. Summary output of PCA model showing the importance of components.

|  | **PC1** | **PC2** | **PC3** | **PC4** |
| --- | --- | --- | --- | --- |
| Standard deviation | 0.6223 | 0.3863 | 0.1643 | 3.411e-16 |
| Proportion of Variance | 0.6873 | 0.2648 | 0.0479 | 0.0000 |
| Cumulative Proportion | 0.6873 | 0.9521 | 1.0000 | 1.0000 |

**Table S4.** Output of PCA model showing the loadings’ contribution to principal components.

|  | **PC1** | **PC2** | **PC3** | **PC4** |
| --- | --- | --- | --- | --- |
| Crushed shell | 0.76393800 | -0.31299746 | 0.2615938 | 0.5 |
| Empty shell | -0.11356935 | 0.79416430 | 0.3261979 | 0.5 |
| Empty shell with operculum | -0.63503190 | -0.51948743 | 0.2772495 | 0.5 |
| Pulled off shell | -0.01533675 | 0.03832059 | -0.8650412 | 0.5 |

**Table S5.** Summary table showing snail condition from the final Generalised Linear Mixed Model using Markov chain Monte Carlo.

|  | **Post mean** | **l-95% CI** | **u-95% CI** | **Eff samp** | **pMCMC** |  |
| --- | --- | --- | --- | --- | --- | --- |
| (Intercept) | -19.39 | -127.86 | 89.62 | 86.694 | 0.6950 |  |
| Site (Tanjong) | 18.19 | -101.49 | 136.21 | 96.697 | 0.7388 |  |
| Site (Pulau Hantu) | -144.83 | -545.11 | 253.78 | 175.186 | 0.4325 |  |
| Site (St John’s) | -165.46 | -515.51 | 241.62 | 205.921 | 0.3525 |  |
| ShoreHeight (Low) | 64.93 | -77.08 | 222.80 | 451.473 | 0.3762 |  |
| Colour (Mixed) | 92.73 | -66.42 | 253.09 | 14.684 | 0.1850 |  |
| Size | 47.84 | -345.55 | 430.72 | 274.315 | 0.8162 |  |
| Site (Tanjong): Shore Height (Low) | -118.18 | -271.58 | 45.20 | 375.070 | 0.1363 |  |
| Site (Pulau Hantu): Shore Height (Low) | 170.51 | -170.66 | 536.69 | 174.610 | 0.2988 |  |
| Site (St John’s): Shore Height (Low) | 5.03 | -283.64 | 333.63 | 168.121 | 0.9263 |  |
| Site (Tanjong): Colour (Mixed) | -142.24 | -352.20 | 41.17 | 5.879 | 0.1075 |  |
| Site (Pulau Hantu): Colour (Mixed) | 471.52 | -58.16 | 1068.63 | 12.515 | 0.0637 |  |
| Site (St John’s): Colour (Mixed) | 682.51 | 106.23 | 1388.85 | 7.054 | 0.0100 | * |
| Shore Height (Low): Colour (Mixed) | -137.69 | -343.19 | 53.51 | 32.601 | 0.1375 |  |
| Site (Tanjong): Size | -120.71 | -494.78 | 322.93 | 215.987 | 0.5513 |  |
| Site (Pulau Hantu): Size | -17.68 | -262.33 | 214.11 | 489.426 | 0.8775 |  |
| Site (St John’s): Size | 244.94 | -37.11 | 541.44 | 34.887 | 0.0750 |  |
| Shore Height (Low): Size | -113.17 | -536.67 | 348.79 | 909.393 | 0.6038 |  |
| Colour (Mixed): Size | -326.63 | -911.92 | 185.07 | 9.628 | 0.1975 |  |
| Site (Tanjong): Shore Height (Low): Colour (Mixed) | 218.31 | -20.11 | 474.00 | 11.898 | 0.0525 |  |
| Site (Pulau Hantu): Shore Height (Low): Colour (Mixed) | 373.10 | -906.74 | 46.78 | 15.573 | 0.0775 |  |
| Site (St John’s): Shore Height (Low): Colour (Mixed) | -289.97 | -788.75 | 92.30 | 17.118 | 0.1450 |  |
| Site (Tanjong): Shore Height (Low): Size | 220.17 | -248.39 | 682.31 | 426.825 | 0.3237 |  |
| Site (Tanjong): Colour (Mixed): Size | 438.65 | -149.89 | 1127.98 | 4.463 | 0.1350 |  |
| Site (Pulau Hantu): Colour (Mixed): Size | -94.27 | -436.45 | 202.62 | 46.066 | 0.5550 |  |
| Site (St John’s): Colour (Mixed): Size | -468.18 | -953.77 | -61.98 | 21.389 | 0.0163 | * |
| Shore Height (Low): Colour (Mixed): Size | 346.30 | -252.13 | 941.36 | 18.121 | 0.2138 |  |
| Site (Tanjong): Shore Height (Low): Colour(Mixed): Size | -482.34 | -1191.72 | 185.16 | 8.701 | 0.1313 |  |
